# Special Issue “Metabolic Syndrome: New Insights in Pathogenesis, Diagnosis, Prevention, and Management”

**DOI:** 10.3390/ijms27156541

**Published:** 2026-07-23

**Authors:** Lucia Maria Procopciuc, Adriana Corina Hangan, Roxana Liana Lucaciu

**Affiliations:** 1 Department of Medical Biochemistry, Faculty of Medicine, “Iuliu Hațieganu” University of Medicine and Pharmacy, 400349 Cluj-Napoca, Romania; 2 Department of Inorganic Chemistry, Faculty of Pharmacy, “Iuliu Hațieganu” University of Medicine and Pharmacy, 400012 Cluj-Napoca, Romania; adriana.hangan@umfcluj.ro; 3 Department of Pharmaceutical Biochemistry and Clinical Laboratory, Faculty of Pharmacy, “Iuliu Hațieganu” University of Medicine and Pharmacy, 400349 Cluj-Napoca, Romania; liana.lucaciu@umfcluj.ro

## 1. Introduction

Metabolic syndrome (MetS) represents one of the most important global health challenges of the twenty-first century. Characterized by the coexistence of central obesity, insulin resistance, dyslipidemia, hypertension, and impaired glucose metabolism, MetS substantially increases the risk of type 2 diabetes mellitus (T2DM), cardiovascular disease, metabolic dysfunction-associated steatotic liver disease (MASLD), chronic kidney disease, and premature mortality [1,2,3,4]. The prevalence of MetS has increased dramatically over recent decades, paralleling the worldwide epidemic of obesity and sedentary lifestyles [1,2]. Current estimates suggest that approximately one-quarter of the global adult population fulfills diagnostic criteria for MetS, with prevalence continuing to rise across both developed and developing countries [1,2,5]. Particularly concerning is the increasing incidence of obesity and metabolic abnormalities among children and adolescents, indicating that the burden of metabolic diseases will likely continue to escalate in future generations [5,6].

Although MetS is traditionally defined through a cluster of clinical and biochemical abnormalities, its pathophysiology extends far beyond the individual diagnostic criteria. Contemporary evidence supports the concept that MetS is a systemic disorder driven by complex interactions among genetic predisposition, environmental exposures, dietary habits, physical inactivity, endocrine disturbances, and aging-related processes [2,7,8]. Central adiposity remains a key pathogenic factor, as dysfunctional adipose tissue acts not only as an energy storage organ but also as an active endocrine and immune organ capable of secreting adipokines, cytokines, chemokines, and extracellular vesicles that influence whole-body metabolism [7,8,9].

Chronic low-grade inflammation has emerged as a hallmark of MetS [7,9,10]. Hypertrophic adipocytes and infiltrating immune cells produce elevated levels of pro-inflammatory mediators, including tumor necrosis factor-alpha (TNF-α), interleukin-6 (IL-6), interleukin-1β (IL-1β), and monocyte chemoattractant protein-1 [9,10,11]. These inflammatory signals interfere with insulin signaling pathways and contribute to the development of systemic insulin resistance. In parallel, oxidative stress further amplifies tissue damage through excessive production of reactive oxygen and nitrogen species, leading to mitochondrial dysfunction, lipid peroxidation, protein modification, and DNA damage. The reciprocal relationship between inflammation and oxidative stress creates a self-perpetuating cycle that promotes metabolic deterioration and end-organ complications [10,11,12].

Mitochondrial dysfunction has gained increasing attention as a central contributor to MetS pathogenesis [11,12,13,14]. Alterations in mitochondrial biogenesis, impaired oxidative phosphorylation, defective mitophagy, and excessive reactive oxygen species production compromise cellular energy homeostasis and exacerbate metabolic abnormalities [12,13,14]. These alterations have been implicated in the progression of obesity, T2DM, MASLD, cardiovascular disease, and other obesity-associated complications. Recent studies have highlighted the importance of mitochondrial quality control mechanisms and immunometabolic interactions as promising therapeutic targets for metabolic diseases [13,14,15].

The growing recognition of genetic and epigenetic contributions has further expanded our understanding of MetS. Genome-wide association studies have identified numerous susceptibility loci associated with obesity, insulin resistance, dyslipidemia, and related metabolic traits [16,17]. At the same time, epigenetic mechanisms—including DNA methylation, histone modifications, and non-coding RNAs—have emerged as important regulators of gene expression involved in lipid metabolism, glucose homeostasis, inflammation, and fibrosis [17,18]. These findings have opened new opportunities for the identification of biomarkers capable of improving risk stratification, early diagnosis, and personalized therapeutic approaches [18].

Another important aspect of contemporary metabolic research concerns developmental programming. Increasing evidence indicates that adverse exposures during fetal life and early postnatal development can induce long-lasting metabolic alterations that predispose individuals to obesity, diabetes, cardiovascular disease, and metabolic syndrome later in life [19,20,21]. Maternal nutrition, environmental stressors, and early-life inflammatory stimuli may influence organ development through epigenetic mechanisms and nutrient-sensing pathways, thereby shaping long-term metabolic health [20,21].

Given the complexity and multidimensional nature of MetS, continuous efforts are required to identify novel biomarkers, clarify pathogenic mechanisms, and develop effective preventive and therapeutic strategies. The Special Issue entitled “Metabolic Syndrome: New Insights in Pathogenesis, Diagnosis, Prevention, and Management” was conceived to address these challenges by bringing together original research articles and comprehensive reviews covering molecular, cellular, animal, and clinical aspects of MetS.

In the opening review of this Special Issue, Lucaciu et al. provide a comprehensive overview of the current understanding of MASLD, one of the most prevalent hepatic manifestations of MetS. The authors emphasize that MASLD should no longer be regarded solely as a liver disease, but rather as a complex multisystem disorder closely linked to obesity, insulin resistance, dyslipidemia, type 2 diabetes mellitus, and cardiovascular disease. Through an extensive analysis of recent literature, the review summarizes the molecular mechanisms underlying hepatic steatosis, inflammation, and fibrosis, highlighting the central roles of insulin resistance, lipotoxicity, oxidative stress, mitochondrial dysfunction, and chronic low-grade inflammation. The authors further discuss current diagnostic approaches, including biochemical markers, imaging techniques, and non-invasive fibrosis assessment tools, while also addressing the limitations of liver biopsy. Particular attention is given to emerging pharmacological therapies targeting different pathogenic pathways involved in MASLD progression. Although no universally approved treatment currently exists, several promising molecules are undergoing advanced clinical evaluation and may significantly alter disease management in the near future. This review establishes an important conceptual framework for the Special Issue by illustrating how MetS affects multiple organs through interconnected molecular pathways. Moreover, it highlights the urgent need for personalized therapeutic approaches capable of simultaneously targeting metabolic dysfunction, inflammation, and fibrosis, which remain major drivers of disease progression and adverse clinical outcomes.

Choi et al. explore one of the central pathogenic mechanisms underlying MetS: the bidirectional relationship between chronic inflammation and mitochondrial dysfunction. In this comprehensive review, the authors examine how obesity-induced adipose tissue expansion promotes the secretion of pro-inflammatory cytokines, including TNF-α, IL-6, and IL-1β, while simultaneously increasing circulating free fatty acids. Together, these alterations contribute to systemic insulin resistance, ectopic lipid accumulation, and progressive metabolic deterioration. A major strength of the review lies in its detailed discussion of mitochondrial dysfunction as both a consequence and a driver of metabolic disease. The authors describe how impaired oxidative phosphorylation, mitochondrial DNA damage, defective mitophagy, and excessive reactive oxygen species production activate inflammatory signaling pathways such as the NLRP3 inflammasome. This creates a vicious cycle in which inflammation and mitochondrial dysfunction perpetuate one another, ultimately promoting β-cell dysfunction, hepatic steatosis, and the development of T2DM. Importantly, the review also highlights potential therapeutic strategies aimed at interrupting this pathogenic loop. Lifestyle interventions, mitochondria-targeted antioxidants, inflammasome inhibitors, and approaches that enhance mitochondrial biogenesis or quality control are discussed as promising avenues for future treatment. The work significantly contributes to the Special Issue by emphasizing the molecular convergence of inflammation and mitochondrial dysfunction as a unifying mechanism linking obesity to multiple metabolic complications.

Lucaciu et al. focus on the emerging role of non-coding RNAs (ncRNAs) in the pathogenesis and progression of nonalcoholic fatty liver disease (NAFLD), a condition that remains one of the most common metabolic disorders worldwide. Given the increasing prevalence of NAFLD and its close association with obesity, insulin resistance, and metabolic syndrome, identifying novel molecular regulators has become a priority for both researchers and clinicians. In this review, the authors comprehensively summarize current evidence regarding the involvement of microRNAs (miRNAs), long non-coding RNAs (lncRNAs), circular RNAs (circRNAs), and PIWI-interacting RNAs (piRNAs) in hepatic lipid metabolism, inflammation, fibrosis, and hepatocarcinogenesis. These epigenetic regulators are shown to influence multiple signaling pathways involved in disease initiation and progression, thereby contributing to the transition from simple steatosis to nonalcoholic steatohepatitis, cirrhosis, and hepatocellular carcinoma. Beyond their mechanistic relevance, the review highlights the potential utility of ncRNAs as minimally invasive biomarkers for early diagnosis, disease monitoring, and therapeutic response assessment. Furthermore, several ncRNA-based therapeutic strategies are discussed, reflecting the growing interest in precision medicine approaches for metabolic liver diseases. This article complements the broader themes of the Special Issue by demonstrating how epigenetic regulation contributes to metabolic dysfunction and by identifying promising molecular targets that may facilitate earlier intervention and more personalized management strategies in patients with NAFLD and MetS.

Rak-Pasikowska et al. present an experimental study investigating whether polyphenols derived from pomegranate peel extract can attenuate oxidative stress and inflammatory alterations associated with MetS. Using Zucker Diabetic Fatty rats, a well-established animal model of obesity and metabolic dysfunction, the authors evaluated the effects of two different doses of pomegranate peel extract on several biochemical, inflammatory, antioxidant, and apoptotic markers within splenic tissue. The study demonstrated that pomegranate polyphenols enhanced antioxidant defense mechanisms by increasing catalase and glutathione peroxidase activities while simultaneously reducing reactive oxygen and nitrogen species levels. These findings support the growing body of evidence indicating that plant-derived polyphenols may counteract the oxidative stress that characterizes MetS. Interestingly, the authors also observed alterations in apoptotic signaling, including reduced BCL-2 gene expression in metabolically affected animals, suggesting that MetS may promote pro-apoptotic pathways within immune tissues. The spleen plays a critical role in immune regulation, yet it remains relatively understudied in the context of MetS. Therefore, this work expands current knowledge by demonstrating that metabolic disturbances extend beyond classical metabolic organs and may affect systemic immune homeostasis. The findings further support the potential use of nutraceutical and dietary interventions rich in polyphenols as adjunctive strategies to mitigate inflammation and oxidative stress in metabolic diseases.

The systematic review and meta-analysis conducted by García-Orozco et al. addresses an increasingly important area of metabolic research: developmental programming. Specifically, the authors investigated whether exposure to early postnatal maternal separation stress influences pancreatic function and metabolic outcomes later in life. Following PRISMA and SYRCLE guidelines, the review synthesized data from 25 experimental studies evaluating pancreatic morphology, glucose metabolism, insulin sensitivity, and glucose tolerance in rodents subjected to maternal separation. The pooled analyses revealed consistent evidence that early-life stress is associated with higher glucose concentrations, impaired glucose tolerance, and reduced insulin sensitivity, as reflected by lower QUICKI scores. These findings indicate that maternal separation can induce long-lasting alterations in pancreatic function and glucose homeostasis, ultimately increasing susceptibility to metabolic dysfunction. Importantly, the results support the concept that environmental exposures during critical developmental windows may shape disease risk long before the appearance of clinical manifestations. This study broadens the scope of metabolic syndrome research by emphasizing that disease susceptibility is not solely determined by adult lifestyle factors. Instead, metabolic vulnerability may originate during early developmental stages through mechanisms involving neuroendocrine dysregulation, chronic stress responses, inflammation, and epigenetic modifications. The review, therefore, provides compelling evidence supporting the developmental origins of health and disease paradigm and highlights the importance of preventive interventions targeting maternal and early-life environments.

In their comprehensive scoping review, Tain and Hsu examine the increasingly recognized concept that maternal nutrition plays a pivotal role in shaping long-term cardiovascular, renal, and metabolic health in offspring. Building upon the developmental origins of health and disease (DOHaD) hypothesis, the authors explore how nutritional exposures during critical periods of fetal development can influence the risk of cardiovascular–kidney–metabolic syndrome later in life. Given the growing prevalence of obesity, diabetes, hypertension, and chronic kidney disease worldwide, identifying early preventive strategies has become a major research priority. The review synthesizes evidence from both experimental and clinical studies investigating various maternal dietary interventions, including amino acid supplementation, lipid-modified diets, micronutrients, gut microbiota-targeted approaches, and plant-derived polyphenols. The authors discuss how these interventions may modulate nutrient-sensing pathways, oxidative stress responses, inflammatory signaling, and epigenetic regulation, thereby influencing fetal programming and disease susceptibility. Importantly, several dietary strategies demonstrated beneficial effects on multiple cardiovascular–kidney–metabolic components simultaneously, suggesting that maternal nutrition may represent a powerful preventive tool against future metabolic disorders. This review expands the traditional understanding of metabolic syndrome by emphasizing that disease risk may originate long before adulthood. By highlighting the interaction between maternal nutrition and developmental programming, the study reinforces the importance of preventive approaches targeting early life stages and provides a valuable framework for future translational research aimed at reducing the global burden of cardiometabolic disease.

Podeanu et al. investigated the complex relationship between obesity, MetS, iron metabolism, and hepatic alterations in a pediatric population, with particular emphasis on the potential role of serum ferritin as an inflammatory biomarker. In this retrospective study involving 68 children and adolescents, the authors evaluated MetS components, serum iron and ferritin concentrations, and liver ultrasonographic findings to identify markers associated with metabolic dysfunction at an early age. The results revealed a markedly higher prevalence of MetS among obese children compared with normal-weight individuals. Furthermore, ferritin concentrations increased progressively with the number of MetS components, whereas serum iron levels declined. Notably, hepatic steatosis detected by ultrasound was strongly associated with elevated ferritin levels and iron deficiency, suggesting a close interaction between inflammation, iron homeostasis, and liver involvement in pediatric MetS. These findings are particularly relevant given the increasing prevalence of childhood obesity worldwide. Ferritin is traditionally considered a marker of iron stores; however, accumulating evidence indicates that it also functions as an acute-phase reactant reflecting systemic inflammation. The study therefore supports the growing concept that low-grade inflammation is already present during the early stages of MetS development. By identifying ferritin as a potential marker of metabolic and hepatic complications, this work may contribute to improved risk stratification and earlier identification of children at increased risk of future cardiometabolic disease.

Hertiš Petek et al. explored the relationship between vitamin D status, inflammation, oxidative stress, and metabolic syndrome in children and adolescents with overweight or obesity. Although vitamin D deficiency has frequently been linked to obesity and metabolic disorders, its precise role in the inflammatory and oxidative pathways underlying MetS remains incompletely understood. To address this question, the authors evaluated a broad panel of anthropometric, biochemical, inflammatory, and oxidative stress markers in a cohort of eighty pediatric participants. As expected, children with overweight and obesity exhibited lower circulating vitamin D concentrations together with increased blood pressure, greater visceral and subcutaneous adiposity, and elevated inflammatory markers, including leukocyte counts, C-reactive protein, and myeloperoxidase. Moreover, individuals meeting criteria for MetS displayed reduced adiponectin concentrations, further supporting the presence of metabolic dysregulation. Interestingly, despite significant correlations between vitamin D levels and adiposity indices, mediation and regression analyses failed to demonstrate a direct relationship between vitamin D status and inflammatory or oxidative stress biomarkers. These findings challenge the widely held assumption that vitamin D deficiency is a major driver of obesity-related inflammation. Instead, the results suggest that excess adiposity itself may represent the primary determinant of inflammatory and oxidative abnormalities in pediatric MetS. By carefully dissecting these relationships, the study contributes valuable evidence to an ongoing debate and highlights the need for a more nuanced understanding of vitamin D biology within the broader context of metabolic disease.

Hernández-Díaz et al. performed a systematic review and meta-analysis to clarify the relationship between circulating leptin levels and metabolic disorders, particularly obesity and diabetes mellitus. Although leptin is widely recognized as a key regulator of appetite, energy expenditure, and body weight homeostasis, previous studies have reported inconsistent findings regarding its clinical significance in metabolic disease. To address these discrepancies, the authors systematically evaluated available evidence from multiple databases and synthesized data from fifteen studies, including 1388 patients and 3536 controls. The meta-analysis demonstrated significantly elevated serum and plasma leptin concentrations in individuals with diabetes compared with healthy controls. Similarly, leptin levels were substantially increased among patients with obesity, supporting the concept of leptin resistance as a hallmark of metabolic dysfunction. Importantly, these associations remained evident across different populations and sex-specific analyses, reinforcing the robustness of the findings. The study provides important support for the use of leptin as a biomarker of metabolic disturbance and highlights its role as a central mediator linking adiposity to insulin resistance and cardiometabolic complications. Although additional studies are required to establish standardized clinical applications, the findings reinforce the growing recognition that adipokines are not merely markers of adipose tissue mass but active participants in the pathogenesis of MetS. Consequently, leptin signaling pathways may represent attractive targets for future therapeutic interventions.

Mitroi Sakizlian et al. investigated the relationship between cardiometabolic phenotypes and surrogate markers of cardiovascular risk in individuals with prediabetes and T2DM. Recognizing that body mass index (BMI) alone inadequately reflects metabolic health, the authors focused on metabolically unhealthy normal-weight and metabolically unhealthy obese phenotypes, assessing their associations with several emerging cardiovascular risk indices, including the triglyceride–glucose (TyG) index, atherogenic index of plasma, cardiometabolic index, and cardiac risk ratio. The cross-sectional study included 300 participants and demonstrated that individuals classified as metabolically unhealthy obese consistently exhibited higher cardiometabolic risk scores than those with metabolically unhealthy normal-weight phenotypes. Furthermore, patients with established T2DM showed more pronounced alterations than prediabetic subjects. Particularly noteworthy were the strong correlations observed between TyG-derived indices and anthropometric measures such as BMI, waist-to-hip ratio, waist-to-height ratio, and body fat percentage. The findings support the emerging concept that metabolic health status may be more clinically relevant than body weight alone when evaluating cardiovascular risk. This distinction is particularly important because substantial heterogeneity exists among individuals with similar body mass indices. By highlighting the utility of novel cardiometabolic indices for early risk stratification, the study contributes to the development of more personalized approaches to MetS management and reinforces the importance of integrating biochemical and anthropometric markers in routine clinical practice.

Chou et al. investigated the relationship between circulating YKL-40 levels, insulin resistance, and MetS in a large cohort of individuals without diabetes. YKL-40, also known as chitinase-3-like protein 1, has emerged as a potential inflammatory biomarker associated with obesity, endothelial dysfunction, and cardiometabolic disorders; however, its precise relationship with MetS remains incompletely understood. To address this issue, the authors analyzed data from 4303 participants enrolled in the Taiwan Biobank, using TyG-BMI as a surrogate marker of insulin resistance and established AHA/NHLBI criteria for MetS diagnosis. The study demonstrated significant positive associations between YKL-40 concentrations and TyG-BMI-derived insulin resistance. Importantly, these relationships persisted even after adjustment for traditional metabolic risk factors, including waist circumference and obesity status. Furthermore, increasing YKL-40 quartiles were associated with a progressively higher prevalence of MetS in both normal-weight and overweight individuals. Interestingly, interaction analyses suggested that the relative contribution of obesity diminished at higher YKL-40 levels, highlighting the independent contribution of inflammatory pathways to metabolic dysfunction. These findings support the growing recognition that chronic inflammation represents a central driver of MetS beyond excess adiposity alone. By identifying YKL-40 as an independent predictor of insulin resistance and MetS, this study expands the repertoire of emerging biomarkers with potential utility for early risk assessment and personalized prevention strategies. Moreover, the results reinforce the concept that inflammatory processes may precede overt diabetes and contribute to disease development long before clinical manifestations become evident.

In this large cross-sectional study, Choi et al. explored the relationship between glycemic status and oral microbial composition in a South Korean population. Increasing evidence suggests that the oral microbiome contributes not only to local oral diseases but also to systemic inflammatory and metabolic disorders. The authors therefore investigated whether blood glucose levels influence the prevalence and distribution of oral pathogens associated with periodontitis and dental caries. Using real-time multiplex PCR, the study evaluated a broad spectrum of oral microorganisms in individuals recruited from seventeen health promotion centers across South Korea. The results demonstrated significant differences in microbial composition according to age, sex, and glycemic status. Individuals with diabetes exhibited a higher prevalence of several periodontitis-associated pathogens, including *Aggregatibacter actinomycetemcomitans* and members of the red and orange bacterial complexes. Additionally, *Streptococcus mutans*, a key cariogenic species, was more abundant among diabetic participants, while protective bacterial populations such as *Streptococcus sanguinis* were reduced. The study provides further evidence supporting a bidirectional relationship between metabolic and oral health. Hyperglycemia may alter the oral microenvironment, favoring the growth of pathogenic microbial communities that contribute to chronic inflammation and tissue destruction. Conversely, periodontal inflammation may exacerbate systemic insulin resistance through inflammatory mediators. By highlighting the oral microbiome as a potential component of MetS pathophysiology, this work broadens the current understanding of host–microbe interactions in cardiometabolic disease and suggests new avenues for preventive and therapeutic interventions.

Huang et al. provide a comprehensive review of the role of sphingolipid metabolism in Hashimoto’s thyroiditis, emphasizing the emerging importance of the sphingosine kinase/sphingosine-1-phosphate (SPHK/S1P) signaling pathway in autoimmune and inflammatory processes. Although Hashimoto’s thyroiditis is traditionally considered an endocrine disorder, growing evidence suggests substantial overlap between metabolic dysfunction, immune dysregulation, chronic inflammation, and tissue remodeling. The authors discuss how sphingosine-1-phosphate functions as a bioactive lipid mediator capable of regulating immune cell trafficking, cytokine production, apoptosis, fibrosis, and cellular differentiation. Aberrant activation of the SPHK/S1P/S1PR axis appears to promote persistent Th1-mediated inflammation, STAT3 activation, epithelial–mesenchymal transition, extracellular matrix remodeling, and fibrotic progression. Moreover, dysregulated S1P signaling may contribute to the increased risk of papillary thyroid carcinoma observed in patients with Hashimoto’s thyroiditis. Although not directly focused on MetS, this review is highly relevant to the broader themes of the Special Issue. Sphingolipids have emerged as critical mediators linking metabolism, inflammation, and immune responses, and alterations in sphingolipid signaling have also been implicated in obesity, insulin resistance, atherosclerosis, and fatty liver disease. Consequently, the review provides valuable insights into shared molecular pathways that may connect autoimmune diseases with metabolic disorders, highlighting novel targets for future therapeutic development.

De Melo et al. focus on the AGPAT2 gene, a critical regulator of adipocyte differentiation and triglyceride biosynthesis whose dysfunction underlies congenital generalized lipodystrophy type 1 (CGL1). Although CGL1 is a rare monogenic disorder, it provides a unique human model for understanding the fundamental biological role of adipose tissue in metabolic homeostasis. Individuals with AGPAT2 mutations exhibit near-complete absence of adipose tissue and develop severe metabolic abnormalities, including insulin resistance, diabetes, hypertriglyceridemia, hypoleptinemia, and hepatic steatosis. The review integrates molecular, genetic, and clinical evidence regarding the pathogenic variants identified in AGPAT2 and evaluates their impact on protein structure, membrane topology, and biological function. Through genotype–phenotype correlations, the authors highlight the considerable clinical heterogeneity observed among affected individuals and discuss how different mutations may influence disease severity. This article contributes significantly to the Special Issue by illustrating the essential role of adipose tissue as a metabolic organ rather than merely an energy reservoir. The severe metabolic disturbances observed in lipodystrophy reinforce the concept that healthy adipose tissue is necessary for maintaining insulin sensitivity and lipid homeostasis. Furthermore, insights derived from rare genetic disorders often provide important mechanistic clues relevant to more common metabolic diseases, including obesity and MetS. Consequently, AGPAT2-related lipodystrophy serves as an informative model for understanding the molecular determinants of metabolic dysfunction.

In the final original research article of this Special Issue, Lynskey et al. investigate the molecular relationship between MetS and end-stage shoulder osteoarthritis using a transcriptomic approach. Although osteoarthritis has traditionally been viewed as a degenerative mechanical disorder, increasing evidence supports the existence of a metabolic phenotype characterized by chronic inflammation, oxidative stress, and altered cellular metabolism. The authors therefore sought to determine how MetS influences gene expression patterns in patients with primary glenohumeral osteoarthritis and cuff-tear arthropathy. Using RNA sequencing of synovial tissue, joint capsule, and subchondral bone samples obtained from patients undergoing shoulder surgery, the investigators identified distinct transcriptomic signatures associated with each pathological condition. Cuff-tear arthropathy was characterized by mitochondrial dysfunction, altered WNT/β-catenin signaling, and enhanced cartilage degradation, whereas primary osteoarthritis exhibited stronger inflammatory activation, complement pathway upregulation, and extracellular matrix remodeling. Importantly, MetS amplified these pathological changes through mechanisms involving oxidative stress, advanced glycation end products, mitochondrial impairment, and extracellular matrix disruption. The study highlights the increasingly recognized concept that metabolic syndrome contributes to musculoskeletal disease through systemic molecular mechanisms. Furthermore, the identification of candidate biomarkers such as ACAN, PANX3, CLU, and VAT1L provides valuable insights into disease pathogenesis and may facilitate the development of targeted therapies. By extending the impact of metabolic syndrome beyond traditional cardiometabolic organs, this work underscores the systemic nature of metabolic dysfunction and concludes the Special Issue with a highly translational perspective on the broad consequences of metabolic disease.

## 2. Conclusions and Future Perspectives

The contributions included in this Special Issue provide a comprehensive and multidisciplinary overview of current advances in MetS research, highlighting the remarkable complexity of this condition and its systemic consequences. Collectively, the studies reinforce the concept that MetS should not be viewed merely as a cluster of metabolic abnormalities but rather as a multifactorial and multisystem disorder driven by intricate interactions among genetic susceptibility, epigenetic regulation, inflammation, oxidative stress, mitochondrial dysfunction, environmental exposures, and lifestyle factors.

A recurring theme across many of the articles is the central role of chronic low-grade inflammation in metabolic disease pathogenesis. The reviews by Choi et al. and Lucaciu et al., together with the original studies investigating ferritin, YKL-40, and leptin, demonstrate that inflammatory pathways contribute not only to insulin resistance and obesity but also to the development of MASLD, cardiovascular disease, and other metabolic complications. These findings support the growing recognition that inflammation is both a consequence and a driver of metabolic dysfunction, creating self-perpetuating pathological loops that accelerate disease progression. In parallel, oxidative stress and mitochondrial impairment emerged as fundamental mechanisms linking excess nutrient availability, altered energy metabolism, and tissue injury. The identification of mitochondrial dysfunction as a common denominator across obesity, diabetes, MASLD, and even osteoarthritis underscores its potential as a promising therapeutic target.

Another important theme addressed throughout this Special Issue concerns the identification of novel biomarkers capable of improving risk stratification and early diagnosis. Ferritin, leptin, YKL-40, and several transcriptomic and microbiome-derived markers were shown to provide valuable information regarding metabolic risk and disease progression. Furthermore, the growing interest in non-coding RNAs highlights the increasing importance of epigenetic biomarkers in metabolic medicine. Advances in transcriptomics, metabolomics, and systems biology are expected to accelerate the discovery of clinically useful biomarkers that may facilitate precision medicine approaches and enable earlier interventions before irreversible organ damage develops.

The articles devoted to developmental programming provide compelling evidence that susceptibility to metabolic disease may originate long before adulthood. Both maternal dietary exposures and early-life stress were shown to influence long-term metabolic outcomes through mechanisms involving nutrient-sensing pathways, neuroendocrine regulation, and epigenetic modifications. These findings support the developmental origins of health and disease paradigm and emphasize the importance of preventive strategies targeting pregnancy, infancy, and childhood. Given the alarming rise in pediatric obesity and MetS worldwide, interventions implemented during these critical developmental windows may have profound implications for reducing future disease burden.

Several contributions also expanded the traditional boundaries of MetS research by examining conditions not classically considered part of the metabolic spectrum. The studies addressing oral microbiota composition, Hashimoto’s thyroiditis, congenital generalized lipodystrophy, and shoulder osteoarthritis demonstrate how metabolic dysfunction influences diverse biological systems through shared molecular pathways. These observations further support the emerging view that MetS is a systemic disorder affecting multiple organs and tissues through common mechanisms involving inflammation, immune dysregulation, lipid signaling, extracellular matrix remodeling, and mitochondrial dysfunction.

From a translational perspective, the studies included in this Special Issue identify several promising therapeutic opportunities. Lifestyle interventions remain the cornerstone of MetS management; however, accumulating evidence suggests that future treatment strategies will increasingly target specific molecular pathways. Potential approaches include therapies directed at mitochondrial quality control, inflammasome regulation, oxidative stress reduction, epigenetic modulation, adipokine signaling, gut microbiota composition, and sphingolipid metabolism. Moreover, advances in MASLD therapeutics and the development of novel biomarker-guided interventions may contribute to more individualized and effective treatment strategies.

Despite substantial progress, many important questions remain unanswered. Future studies should focus on validating emerging biomarkers in large, ethnically diverse populations and determining their clinical utility in routine practice. Longitudinal investigations are needed to clarify causal relationships among inflammation, mitochondrial dysfunction, microbiome alterations, and metabolic disease progression. Further research should also explore the integration of multi-omics technologies, including genomics, transcriptomics, epigenomics, proteomics, and metabolomics, to generate a more comprehensive understanding of disease mechanisms. Additionally, greater attention should be devoted to developmental programming, sex-specific differences, and the interactions between genetic susceptibility and environmental exposures throughout the life course.

Taken together, the articles presented in this Special Issue illustrate the rapid evolution of MetS research from descriptive clinical observations toward a deeper mechanistic understanding of disease biology. By integrating insights from molecular biology, experimental models, epidemiology, clinical medicine, and translational research, these contributions significantly advance our understanding of the pathogenesis, diagnosis, prevention, and management of MetS. Ultimately, continued interdisciplinary efforts will be essential to translate these discoveries into effective strategies capable of reducing the growing global burden of metabolic diseases and improving long-term patient outcomes.

## Data Availability

No new data were created or analyzed in this study. Data sharing is not applicable to this article.

## Abbreviations

The following abbreviations are used in this manuscript:

BMI	Body mass index
IL-6/IL-1β	Interleukin-6/Interleukin-1β
MASLD	Metabolic dysfunction-associated steatotic liver disease
MetS	Metabolic syndrome
NAFLD	Nonalcoholic fatty liver disease
ncRNAs	Non-coding RNAs
T2DM	Type 2 diabetes mellitus
TNF-α	Tumor necrosis factor-alpha
TyG	Triglyceride-glucose
